# Graves' Disease and Treatment Effects on Warfarin Anticoagulation

**DOI:** 10.1155/2014/292468

**Published:** 2014-06-04

**Authors:** Amanda Howard-Thompson, Alexis Luckey, Christa George, Beth A. Choby, Timothy H. Self

**Affiliations:** ^1^Department of Clinical Pharmacy and Family Medicine, University of Tennessee Health Science Center, 881 Madison Avenue, Memphis, TN 38163, USA; ^2^Jackson-Madison County General Hospital, 620 Skyline Drive, Jackson, TN 38301, USA; ^3^Department of Medical Education, University of Tennessee Health Science Center, Room 1002, 910 Madison Avenue, Memphis, TN 38163, USA; ^4^Department of Clinical Pharmacy, University of Tennessee Health Science Center, 881 Madison Avenue, Memphis, TN 38163, USA

## Abstract

*Background*. Hyperthyroidism causes an increased hypoprothrombinemic response to warfarin anticoagulation. Previous studies have demonstrated that patients with hyperthyroidism require lower dosages of warfarin to achieve a therapeutic effect. As hyperthyroidism is treated and euthyroidism is approached, patients may require increasing warfarin dosages to maintain appropriate anticoagulation. We describe a patient's varying response to warfarin during treatment of Graves' disease. *Case Presentation*. A 48-year-old African American female presented to the emergency room with tachycardia, new onset bilateral lower extremity edema, gradual weight loss, palpable goiter, and generalized sweating over the prior 4 months. She was admitted with Graves' disease and new onset atrial fibrillation. Primary stroke prophylaxis was started using warfarin; the patient developed a markedly supratherapeutic INR likely due to hyperthyroidism. After starting methimazole, her free thyroxine approached euthyroid levels and the INR became subtherapeutic. She remained subtherapeutic over several months despite steadily increasing dosages of warfarin. Immediately following thyroid radioablation and discontinuation of methimazole, the patient's warfarin dose and INR stabilized. *Conclusion*. Clinicians should expect an increased response to warfarin in patients with hyperthyroidism and close monitoring of the INR is imperative to prevent adverse effects. As patients approach euthyroidism, insufficient anticoagulation is likely without vigilant follow-up, INR monitoring, and increasing warfarin dosages.

## 1. Background

Numerous drugs and diseases affect patients' anticoagulant response to warfarin [1, 2]. The effect of thyroid function on anticoagulation has been a topic of research for many years. Although thyroid function may influence the disposition of many drugs, its effect on warfarin activity is unique. Therefore, patients with thyroid dysfunction should be monitored carefully when receiving warfarin.

Hypothyroidism is associated with a decreased response to warfarin, likely due to decreased catabolism of vitamin K-dependent clotting factors. One case report described a hyperthyroid patient treated with radioactive iodine. This patient was subsequently admitted to the hospital two years later with hypothyroidism and pulmonary embolism requiring 27.5 mg of warfarin daily to maintain a therapeutic range. The warfarin dose requirement was decreased to 10 mg once she achieved euthyroidism [3]. Other similar cases have demonstrated an increased warfarin dosage requirement for anticoagulation when hypothyroidism is present, with a decreased dosage requirement once normal thyroid function is attained [4].

Hyperthyroidism has been shown to increase sensitivity to the hypoprothrombinemic effect of warfarin, requiring lower dosages of the drug for therapeutic effect [5, 6]. Hyperthyroidism causes an increase in both the production and catabolism of vitamin K-dependent clotting factors (II, VII, IX, and X) [7]. Anticoagulation with warfarin decreases the carboxylation of these clotting factors, which reduces their coagulant activity. When a patient with hyperthyroidism is anticoagulated with warfarin, vitamin K-dependent clotting factor production decreases while catabolism of the clotting factors increases. This overall decrease in available clotting factors causes the enhanced warfarin sensitivity noted in patients with hyperthyroidism [8].

Reports demonstrate the need for smaller dosages of warfarin when a patient is in a hyperthyroid state; as thyroid hormone-lowering therapy is initiated and euthyroid hormone levels are approached, higher dosages of warfarin may be needed to maintain a therapeutic international normalized ratio (INR) [4, 9–12]. Despite a few reports of the influence of thyroid dysfunction on the response to warfarin, this drug-disease interaction is easily overlooked, placing the patient at increased risk for poor outcomes. Patients are also often incorrectly counseled that the difficulty stabilizing their anticoagulation therapy is due to a drug-drug interaction between warfarin and the antithyroid agent or thyroid supplement, when the actual cause is uncontrolled thyroid disease. The following case describes a patient's variable response to warfarin while receiving antithyroid pharmacologic therapy and radioactive iodine ablation for Graves' disease. To our knowledge no other case published to date describes the effect of medication therapy and radioactive iodine ablation treatment for Graves' disease on warfarin anticoagulation in such detail.

## 2. Case Presentation

A 48-year-old African American female presented to the emergency department stating that she was “looking for a cure for thyroid disease.” She also noted a 2-day history of new onset bilateral lower extremity edema, gradual weight loss, and generalized sweating. She had been experiencing these symptoms for approximately 4 months. Additionally, she complained of palpitations and mild sinus tachycardia (rate 106) was noted during her initial presentation. Surgical history was significant for cervical cancer status after hysterectomy 2 years prior. She denied taking any medications or any additional medical diagnoses. Social history was significant for tobacco use, although she said she quit smoking three weeks earlier. Family history was significant for two sisters, one brother, and father with Graves' disease. Significant laboratory results included thyroid stimulating hormone (TSH) 0.006 *μ*IU/mL (reference range 0.450–4.500 *μ*IU/mL) and free thyroxine (T_4_) > 7.77 ng/dL (reference range 0.82–1.77 ng/dL). The patient's cardiac enzymes were within normal limits except for a pro-BNP of 3666 pg/mL (reference range 0–125 pg/mL). Serum potassium concentration was 3.0 mEq/L (reference range 3.5–5.1 mEq/L). Liver function tests and serum creatinine were normal. A baseline INR was also obtained (Table 1).

The patient was admitted and was given one dose of both aspirin 325 mg and potassium chloride 40 mEq. Graves' disease was later confirmed by radioactive iodine uptake and a thyroid scan that revealed a markedly elevated 493 uCi I-123 24 hour uptake of 99% (normal range is typically 10–30%). An ultrasound of the soft tissue of her head and neck showed diffuse thyroid enlargement with the right lobe measuring 6 × 3 × 2.5 cm and the left lobe measuring 6.3 × 3 × 2.4 cm. No discrete mass was visible and increased blood flow was noted. She was diagnosed with Graves' disease with a nonvisible, palpable goiter. In addition, microcytic, hypochromic anemia, new onset atrial fibrillation (AF), and diastolic dysfunction with a mildly enlarged right atrium (EF 55–60% on echocardiogram) were also diagnosed.

Primary stroke prophylaxis with warfarin 5 mg daily (with an INR goal range of 2-3) was initiated due to AF with a CHA_2_DS_2_-VASc score of one and a CHADS_2_ score of zero. It was uncertain whether the AF was a preexisting condition or solely due to Graves' disease. Due to the unknown length of time in this irregular rhythm and the young age of the patient with no concurrent disease states or medications, the inpatient medical team decided to start warfarin therapy until her hyperthyroidism was controlled and any underlying cardiac problems were ruled out. Table 1 lists INR results during hospitalization. An increase in INR by 0.2 to 0.3 per day during warfarin initiation is considered an appropriate change in a hospital setting [13]. The patient's INR increased at a rate of 0.6 per day between Day 2 and Day 5, which could be explained by the hyperthyroid state because there was an absence of other known contributing factors. Assuming a rate of INR increase of 0.2 to 0.3 per day, one would have estimated an INR on Day 5 of 2.77 at the highest in a patient without a thyroid disorder. After a one-week inpatient stay, the patient was discharged on warfarin 5 mg daily, metoprolol 12.5 mg twice daily, and benazepril 5 mg daily. The patient was not initiated on any medication while in the hospital to treat her hyperthyroidism. Methimazole 10 mg daily was prescribed upon release from the hospital, but the patient was discharged without getting her prescription. In addition, after the patient left the hospital a supratherapeutic INR result (4.41) was reported, which would likely have initiated a warfarin dosage change.

The patient was scheduled for INR follow-up appointments at her primary care office the following day and again five days after hospital discharge but did not show up for these appointments. She stated that she did not understand the importance of these visits. She returned for follow-up at her primary care office seven days after discharge. Her INR was >8 via CoaguChek XS (Roche Diagnostics) point of care testing, which was performed once on each hand. She reported no signs or symptoms of bleeding. Due to the INR being >4.5 the patient was sent to the lab for a confirmatory venipuncture INR. The venipuncture INR was >10 and the patient was instructed to hold all warfarin doses and return to clinic in 2 days. Vitamin K was not administered due to the patient not having any signs and symptoms of bleeding. At the next visit her CoaguChek XS point of care testing INR was 2.8. Warfarin therapy was resumed at 1 mg daily. Due to the patient's exaggerated response to warfarin, presence of anemia, and hyperthyroid state, the warfarin was dosed conservatively and frequent follow-up with her primary care anticoagulation clinic was recommended to the patient. She was provided a handout on vitamin K content per serving of common dietary foods and counseled on maintaining a consistent Vitamin K intake (please go to http://www.PTINR.com/ for the comprehensive list). The patient was extensively counseled on the effects of vitamin K foods on decreasing the INR as she stated she was unaware of this information prior to this initial outpatient visit. She began taking methimazole 10 mg daily on the day of this visit (Table 2).

She returned for follow-up four days later. At this visit, her CoaguChek XS point of care testing INR was 1.3. When questioned about her use of warfarin and methimazole, she explained that the dispensing pharmacist at her local pharmacy informed her that warfarin reacted with methimazole. Subsequently, the patient's family members advised her not to take the warfarin. The clinical pharmacist managing her anticoagulation at the primary care clinic explained that she needed to take both medications. The patient was informed that methimazole would treat her Graves' disease and that the status of this disease would influence her response to warfarin therapy. The need for close follow-up was reemphasized at this visit and laboratory values were obtained showing a low serum iron (34 *μ*g/dL; reference range 35–155 *μ*g/dL) and low iron saturation (13%; reference range 15–55%). Ferrous sulfate 325 mg three times daily was prescribed. The patient attended multiple follow-up visits over the next four months. During this time her free T_4_ levels decreased to euthyroid levels. Her weight increased by 14 pounds. Her INR was subtherapeutic at almost every visit, despite increasing dosages of warfarin (Table 2).

After four months of methimazole therapy, the patient was scheduled for radioactive iodine ablation by her endocrinologist. Two weeks after this procedure, her CoaguChek XS point of care testing INR was 3.1. She had experienced one episode of epistaxis lasting 4 minutes a few days prior to the INR test but was able to stop the bleeding. She also reported slightly less vitamin K consumption due to decrease in dietary intake. She was advised to maintain consistent vitamin K intake and to decrease her dose from 42.5 mg/week to 40 mg/week. The patient did not decrease the dose but remained on the 42.5 mg/week dose. She returned to the clinic 10 days later with a CoaguChek XS point of care testing INR of 2.9. After methimazole was discontinued and the thyroid gland was ablated, the patient only required 1 dose adjustment for a supratherapeutic INR (Table 3). Levothyroxine 75 mcg daily was started 3 months after ablation. The patient described intermittent adherence to her levothyroxine therapy. The endocrinologist decided to discontinue the levothyroxine based on laboratory values showing a decreased TSH and normal to elevated free T_4_ serum concentration (Table 4), indicating induction of a hyperthyroid state from the administration of levothyroxine. Despite this, the patient did not require a warfarin dose adjustment and only had one subtherapeutic INR during this time. When levothyroxine was stopped, electrocardiograms done at both her PCP and cardiologist's offices showed normal sinus rhythm. Due to patient's symptoms resolving with treatment and the normal EKG results, warfarin therapy was discontinued.

## 3. Discussion

Interestingly another antithyroid agent, propylthiouracil (PTU) has been described in the literature to cause hypoprothrombinemia when used as monotherapy for hyperthyroidism. A patient was reported to have received PTU and was admitted to the hospital with gingival bleeding, prolonged metrorrhagia, and severe hypoprothrombinemia after one year of treatment [14]. This patient was also reported to have a blood diathesis, which could have contributed to the adverse effect, but no lab values or further description of the blood disorder was provided.

In 1962, Owens et al. investigated dextrothyroxine's effect on the clotting mechanism after observing that anticoagulated patients receiving this agent tended to require lower than usual anticoagulant dosages [15]. Dextrothyroxine is the stereoisomer of L-thyroxine, an antithyroid agent that is no longer used for the treatment of hyperthyroidism. All patients taking dextrothyroxine while receiving stable, long-term anticoagulation had further prolongation of prothrombin time. The authors concluded that the combination of dextrothyroxine and warfarin should only be used with the awareness that dextrothyroxine would potentiate warfarin's anticoagulant effect. They also noted that hemorrhagic complications could be prevented through weekly observation of clotting factor levels and appropriate dosage adjustments during the first month of therapy.

Another study evaluated the effect of hyperthyroidism and warfarin by measuring activity of clotting factors prothrombin (II), VII, VIII, IX, and X, prothrombin ratio (PTR), and partial thromboplastin time with kaolin (PTT-K) in five hyperthyroid patients prior to and then following a single dose of warfarin [16]. These measures were repeated once patients were euthyroid. There was lower activity of factor II and shorter PTT-K in hyperthyroidism compared to euthyroidism. Warfarin administration caused a further decrease in factors II and VII and greater increase in PTR and PTT-K in patients when in a hyperthyroid state. Patients with hyperthyroidism therefore had a more exaggerated response to warfarin anticoagulation.

The first case report of increased response to warfarin in a patient with Graves' disease was described in 1972 [9]. The patient's prothrombin time was therapeutic and stable on warfarin maintenance therapy when in a euthyroid state, but two separate episodes of hyperthyroidism caused a marked increase in prothrombin time. Warfarin dosage was decreased to prevent bleeding. After returning to euthyroid status following both episodes, a therapeutic INR was reached on the initial maintenance dose. Following this case report, there have been other similar clinical observations and a review of this topic [10–12, 17].

The patient described in this case initially had an increased, supratherapeutic response to warfarin administration secondary to hyperthyroidism. After starting methimazole, her free thyroxine approached euthyroid levels and the INR became subtherapeutic. Even with steadily increasing warfarin dosages, she did not maintain a therapeutic INR. However, once the patient received radioactive iodine ablation her dose and INR stabilized despite inducing a slightly hyperthyroid state with levothyroxine supplementation. To our knowledge no one has published the full effects of hyperthyroid treatment (both medication and ablation) on INR results in a patient receiving warfarin therapy.

## 4. Conclusion

Hyperthyroidism causes an increased sensitivity to the anticoagulant effects of warfarin. This may lead to a supratherapeutic INR and possibly bleeding if warfarin dosages are not adjusted cautiously. Monitoring of the anticoagulation response to warfarin is imperative in patients with concomitant hyperthyroidism as increased INRs are probable. Physicians and pharmacists must be aware of the effects of fluctuating thyroid hormone levels and monitor patients regularly in order to appropriately prescribe and counsel patients receiving concomitant thyroid hormone altering therapy and anticoagulation.

## Figures and Tables

**Table 1:** Inpatient warfarin response during hyperthyroid storm.

Day of warfarin	Venopuncture INR^a^	Warfarin dose (mg/day)	TSH^b^ (*μ*IU/mL)	Free T_4_^c^ (ng/dL)
1	1.18^d^	5	0.006	>7.77
2	1.27	5		
5	3.02	5		
7	4.41^e^	5		

^a^INR: international normalized ratio, goals 2-3.

^b^TSH: thyroid stimulating hormone, reference range 0.450–4.500 *μ*IU/mL.

^c^Free T_4_: free thyroxine, reference range 0.82–1.77 ng/dL.

^d^INR was baseline level obtained prior to warfarin dose.

^e^INR result posted after patient had been discharged from hospital.

**Table 2:** Outpatient warfarin response to the addition of methimazole.

Day of methimazole	Warfarin dose (mg/day)^a^	INR^b,c^	New warfarin^a^ dose (mg/day)	Methimazole (mg/day)	TSH^d^ (*μ*IU/mL)	Free T_4_^e^ (ng/dL)
1	Held	2.8	1	10		

4	Patient withheld	1.3	Restart 1 mg	10	<0.005	4.69

12	1	1.1	2	10		

21	2	1.2	3	10	<0.005	1.34

28	3	1.5	4	10	<0.005	1.14

35	4	1.7	5	10		

45	5	2	5	10	<0.005	0.83

63	5	1.6	5: MTWRSSu 7.5: F	10		

81	5: MTWRSSu 7.5: F (missed 1 dose)	1.2	5: TRSSu 7.5: MWF	10	0.007	1.06

112	5: MTWRSSu 7.5: F	1.9	5: TWRSSu 7.5: MF	10	<0.005	0.65

120	Radioactive iodine^131^ ablation					

^a^Letters are abbreviations for days of the week: Monday (M), Tuesday (T), Wednesday (W), Thursday (R), Friday (F), Saturday (S), and Sunday (Su).

^b^INR: international normalized ratio, goals 2-3.

^c^INR obtained via point of care testing with Roche Diagnostics CoaguChek XS.

^d^TSH: thyroid stimulating hormone, reference range 0.450–4.500.

^e^Free T_4_: free triiodothyronine, reference range 0.82–1.77.

**Table 3:** Outpatient warfarin response status after radioactive iodine ablation of the thyroid and discontinuation of methimazole.

Days since ablation	Warfarin dose^a^ (mg/day)	INR^b,c^	New warfarin^a^ dose (mg/day)	TSH^d^ (*μ*IU/mL)	Free T_4_^e^ (ng/dL)	Free T_3_^f^ (pg/mL)
7	5: TWRSSu 7.5: MF	1.6	5: TRSSu 7.5: MWF			

14	5: TThSSu 7.5: MWF	3.1	5: TWThSSu 7.5: MF			

24	5: TWThSSu 7.5: MF	2.9	No change			

42	5: TWThSSu 7.5: MF	3.3	5: MTWThSSu 7.5: F		1.78	4

67	5: MTWThSSu 7.5: F	2.8	No change			

94	5: MTWThSSu 7.5: F	2.7	No change	0.163	0.45	

122	5: MTWThSSu 7.5: F	2.5	No change			

^a^Letters are abbreviations for days of the week: Monday (M), Tuesday (T), Wednesday (W), Thursday (R), Friday (F), Saturday (S), and Sunday (Su).

^b^INR: international normalized ratio, goals 2-3.

^c^INR obtained via point of care testing with Roche Diagnostics CoaguChek XS.

^d^TSH: thyroid stimulating hormone, reference range 0.450–4.500.

^e^Free T_4_: free thyroxine, reference range 0.82–1.77.

^f^Free T_3_: free triiodothyronine, reference range 2–4.4.

**Table 4:** Response to warfarin after levothyroxine initiation.

Days since levothyroxine initiation	Warfarin dose (mg/day)^a^	INR^b,c^	New warfarin^a^ dose (mg/day)	Levothyroxine (75 mcg/day)	TSH^d^ (*μ*IU/mL)	Free T_4_^e^ (ng/dL)	Free T_3_^f^ (pg/mL)
1	5: MTWThSSu 7.5: F	3.2	No change	Not taking			

13	5: MTWThSSu 7.5: F	2.2	No change	Taking since last visit	3.63	0.7	

73	5: MTWThSSu 7.5: F	1.5	No change	Not taking for 2 weeks	<0.006	1.49	

86	5: MTWThSSu 7.5: F	2.8	No change	Only taking for 1week	<0.006	2.11	4.9

133	5: MTWThSSu 7.5: F	2.5	No change	Had not taken since last visit	0.008	1.40	4.2

^a^Letters are abbreviations for days of the week: Monday (M), Tuesday (T), Wednesday (W), Thursday (R), Friday (F), Saturday (S), and Sunday (Su).

^b^INR: international normalized ratio, goals 2-3.

^c^INR obtained via point of care testing with Roche Diagnostics CoaguChek XS.

^d^TSH: thyroid stimulating hormone, reference range 0.450–4.500.

^e^Free T_4_: free thyroxine, reference range 0.82–1.77.

^f^Free T_3_: free triiodothyronine, reference range 2–4.4.
